# The research of the correlations between psychological factors and students’ learning engagement: a systematic review and meta-analysis

**DOI:** 10.3389/fpsyg.2026.1760601

**Published:** 2026-04-21

**Authors:** Shaoshuai Liang, Qian Peng, Qiao Xu, Shaobo Li

**Affiliations:** Shandong College of Traditional Chinese Medicine, Yantai, China

**Keywords:** broaden-and-build theory, meta-analysis, motivation, psychological resilience, self-efficacy, student engagement

## Abstract

**Background:**

Student learning engagement—the cognitive, behavioral, and emotional investment in academic activities—is a key determinant of academic success. Three psychological factors have been identified as predictors: motivation, self-efficacy, and psychological resilience. However, primary studies report inconsistent effect sizes (β = 0.16–0.97) and typically examine these constructs in isolation. No comprehensive meta-analysis has simultaneously evaluated all three predictors’ relative strength within an integrated framework, limiting the empirical basis for designing targeted educational interventions.

**Objective:**

Grounded in the broaden-and-build theory, this systematic review and meta-analysis examines the strength and consistency of associations between motivation, self-efficacy, psychological resilience, and learning engagement, explores their underlying mechanisms, and offers evidence-based recommendations for enhancing psychological support in educational settings.

**Methods:**

Following PRISMA 2020 guidelines, a systematic search was conducted across nine databases (PubMed, Embase, Cochrane, Web of Science, VIP, CNKI, Scopus, PsycINFO, and Ovid) for studies published between January 2018 and June 2025. The protocol was not prospectively registered on PROSPERO or any equivalent platform. Methodological quality was assessed using JBI critical appraisal tools. Standardized path coefficients (β) were extracted and meta-analyses performed using Stata 14.0 and Review Manager 5.4. Heterogeneity, publication bias, and result robustness were evaluated via I^2^, Cochran’s Q, funnel plots, Egger’s test, trim-and-fill adjustments, and sensitivity analyses.

**Results:**

Structural equation modeling (SEM) was the primary analytical method, employed in twenty studies versus five using multiple regression. The meta-analysis revealed statistically significant positive associations of varying magnitude between all three psychological factors and learning engagement. Standardized path coefficients ranged from small-to-medium to large effects: motivation (β = 0.193–0.972), self-efficacy (β = 0.166–0.500), and psychological resilience (β = 0.187–0.539). However, substantial heterogeneity was observed across all three factor groups (*I*^2^ = 81–96%), indicating context-dependent variability in effect magnitudes.

**Conclusion:**

As the first meta-analysis to concurrently examine motivation, self-efficacy, and psychological resilience as predictors of learning engagement, this study demonstrates consistent positive associations across diverse educational contexts. Given the cross-sectional nature of the included studies, findings should be interpreted as associations rather than causal effects. These results provide a robust empirical foundation for developing targeted, psychologically informed educational interventions.

## 1 Introduction

Learning engagement—the degree to which students invest cognitive, behavioral, and emotional effort in academic activities—has emerged as a central construct in educational research (Fredricks et al., 2004). Declining engagement contributes to academic underperformance, increased dropout rates, and diminished wellbeing (Appleton et al., 2008), with PISA data confirming this challenge varies substantially across countries (Organisation for Economic Co-operation and Development [OECD], 2019). In response, multiple nations have initiated policy reforms: China’s 2023 mental health action plan integrates psychological support throughout education (Jiang and Zhang, 2024); the UK mandates whole-school approaches to emotional wellbeing; Australia prioritizes student resilience; and the US CASEL framework embeds social-emotional learning across curricula (Weare, 2015). These converging efforts underscore that psychological factors are foundational to effective learning.

Research consistently identifies motivation, self-efficacy, and psychological resilience as key predictors of learning engagement (Wu and Ma, 2022). Academic motivation—comprising intrinsic and extrinsic forms—serves as the foundational driver of engagement (Setiamurti et al., 2023). Self-efficacy shapes how students approach challenges and sustain effort (Fu et al., 2024), while psychological resilience enables students to maintain persistence and cognitive focus during academic setbacks (Chu et al., 2024). Together, these psychological resources account for approximately 21.9% of the variance in academic engagement among university students (Crisostomus and Saraswati, 2023). Furthermore, resilience directly predicts engagement, with resilient students demonstrating greater active participation (Chu et al., 2024). However, anxiety and low self-belief can substantially hinder engagement (Setiamurti et al., 2023). These findings suggest the three factors carry substantial explanatory power, warranting systematic investigation of their joint contribution.

Although individual contributions of these factors are well-documented, two critical gaps persist. First, existing studies report highly inconsistent effect sizes, ranging from strong associations (β > 0.50; Gavín-Chocano et al., 2024) to weak effects (β < 0.20; Wei et al., 2024). This heterogeneity may stem from differences in measurement instruments, cultural contexts, or analytical methods, but without formal quantitative synthesis, the sources remain unresolved. Second, most studies examine these factors in isolation, neglecting that motivation, self-efficacy, and resilience operate as interconnected psychological resources (Chu et al., 2024). This fragmented approach prevents comparison of relative predictive strength and obscures potential synergistic effects. Existing meta-analyses have similarly focused on single constructs—for example, Li and Xue (2023) examined environmental and behavioral factors without simultaneously assessing all three psychological predictors.

The broaden-and-build theory (Fredrickson, 2001) provides a compelling framework for understanding the synergistic relationship among these factors. We selected this theory over alternatives for specific reasons: Self-Determination Theory (Deci and Ryan, 2000) does not fully account for self-efficacy and resilience; Social Cognitive Theory (Bandura et al., 1999) does not equally address motivation and resilience as parallel resources. The broaden-and-build theory uniquely accommodates all three constructs by explaining how positive psychological resources synergistically accumulate over time. Specifically, positive emotions broaden thought-action repertoires, which build enduring personal resources (Fredrickson, 2001). Applied to education: motivation initiates broadened learning behaviors (Dian-Zhi, 2005); self-efficacy sustains engagement and generates positive effect (Riswantyo and Lidiawati, 2021); resilience prevents resource depletion during setbacks (Kiken and Fredrickson, 2017). Together, these form an upward spiral in which motivated students build efficacy through success, which builds resilience, which sustains deeper engagement.

This study addresses these gaps through a systematic review and meta-analysis simultaneously synthesizing evidence on all three predictors. Guided by the broaden-and-build framework, we aim to: (1) estimate pooled effect sizes for each factor’s association with learning engagement; (2) compare their relative predictive strength; and (3) examine cross-contextual consistency. Random-effects meta-analytic techniques were applied to standardized path coefficients (β) from 25 studies following PRISMA 2020 guidelines.

This study makes several contributions. Theoretically, it is the first meta-analysis to concurrently examine motivation, self-efficacy, and psychological resilience within a unified broaden-and-build framework, enabling direct comparison of effect magnitudes. Practically, by quantifying each factor’s relative strength, it provides empirically grounded evidence to guide intervention prioritization—whether toward motivation strategies, confidence-building programs, or adaptive coping interventions—across diverse educational settings.

## 2 Theoretical framework and hypotheses

The broaden-and-build theory (Fredrickson, 2001) provides the overarching theoretical lens for this meta-analysis, explaining the specific mechanisms through which motivation, self-efficacy, and psychological resilience relate to learning engagement.

Within this framework, intrinsic motivation triggers the broadening process: motivational interest generates positive emotions that broaden thought-action repertoires, manifesting as heightened engagement (Dian-Zhi, 2005). Empirical evidence confirms both intrinsic and extrinsic motivation predict engagement across diverse settings (Setiamurti et al., 2023; Kim and Doo, 2022).

*H1*: Learning motivation is positively associated with students’ learning engagement.

Self-efficacy and learning engagement. Self-efficacy represents a critical “built resource” within the framework. Efficacy beliefs generate positive affective states that broaden engagement repertoires, enabling students to tackle challenging tasks rather than avoiding them, creating an upward spiral where success builds further efficacy (Chen, 2024; Riswantyo and Lidiawati, 2021). Research confirms self-efficacy directly promotes engagement across traditional and online environments (Luo et al., 2023; Pang and Veloo, 2024).

*H2*: Self-efficacy is positively associated with students’ learning engagement.

Self-efficacy works as a mediator. The theory implies that resources built through broadened cognition serve as transmission mechanisms: motivation generates positive affect, broadened engagement leads to mastery experiences, mastery builds self-efficacy, and enhanced efficacy sustains deeper engagement (Wu et al., 2020; Liu et al., 2022; Shao and Kang, 2022).

*H3*: Self-efficacy mediates the relationship between learning motivation and learning engagement.

Psychological resilience and learning engagement. Resilience functions as a protective resource within the broaden-and-build cycle. Academic setbacks trigger negative emotions that narrow thought-action repertoires; resilience enables adaptive recovery through cognitive reappraisal and emotion regulation, preventing sustained narrowing and preserving the upward spiral of resource-building (Chu et al., 2024). Large-scale studies confirm resilience directly predicts engagement (Li et al., 2024; Adeniji et al., 2020).

*H4*: Psychological resilience is positively associated with students’ learning engagement.

Psychological resilience functions as a mediator. The building process suggests motivation fosters resilience as a durable resource: motivated students accumulate adaptive coping strategies that sustain engagement over time (Wang and Zhang, 2024; Gao et al., 2020; Shaoshuai et al., 2025).

*H5*: Psychological resilience mediates the relationship between learning motivation and learning engagement.

While H3 and H5 propose mediating mechanisms, this meta-analysis primarily synthesizes direct associations (H1, H2, H4). The mediation hypotheses are evaluated qualitatively; formal meta-analytic structural equation modeling (MASEM) is recommended for future research.

## 3 Materials and methods

### 3.1 Literature search strategy

Following PRISMA guidelines, a structured electronic search was conducted on January 20, 2025, across nine databases (PubMed, Embase, Cochrane, Web of Science, VIP, CNKI, Scopus, PsycINFO and Ovid). The review protocol was not prospectively registered on PROSPERO or any equivalent platform, which constitutes a limitation. We commit to prospective registration for future systematic reviews and recommend likewise for researchers. The search strategy was as follows: [Title/Abstract] = (“psychological factors” OR “motivation” OR “self-efficacy”) AND [Title/Abstract] = (“learning engagement” OR “cognitive involvement” OR “academic engagement”) AND [Title/Abstract] = (“multiple regression analysis” OR “structural equation modeling” OR “SEM”). The search encompassed peer-reviewed literature published in English or Chinese between January 2018 and June 2025. The 8-year limit is a strategic filter to ensure the review is current, relevant, manageable, and based on the highest quality and most recent evidence available.

### 3.2 Inclusion and exclusion criteria

Inclusion criteria: ➀ Participants aged 12–25 years; ➁ Studies assessing psychological affecting factors; ➂ Studies evaluating students’ learning engagement; ➃ Quantitative examination of the correlation between psychological determinants and learning engagement; ➄ Cross-sectional and longitudinal study methodologies; ➅ Publications in English or Chinese.

Exclusion criteria: (1) Non-peer-reviewed literature; (2) Studies with < 100 participants; (3) Those lacking data on the association between psychological factors and learning engagement; (4) Reviews or studies relying solely on Spearman/Pearson correlation coefficients without further association data; (5) The study focused on the randomized controlled trial. The minimum sample size threshold of 100 was established based on methodological guidelines recommending 100–200 participants for basic SEM models (Kline, 2016). We acknowledge more complex models may require larger samples. Due to limited studies in each subgroup, sensitivity analyses excluding smaller studies (*n* < 200) were not feasible but are recommended for future meta-analyses with larger study pools. This ensured the analysis focused on robust, relevant primary evidence. Peer review is a critical benchmark for methodological rigor, validity, and scientific credibility. The Spearman and Pearson correlation methods focus solely on examining associations between variables, whereas this study is concerned with investigating the relationships among variables. This study aims to delineate the associative relationships among motivation, self-efficacy, psychological resilience, and learning engagement. Randomized Controlled Trials (RCTs) are inherently suited to intervention-based effect evaluation, which falls beyond the scope of this observational inquiry. Similarly, Analysis of Variance (ANOVA) is predominantly employed to compare means across three or more groups—a methodology closely aligned with experimental designs such as RCTs for assessing intervention-related outcomes. By excluding both RCTs and ANOVA-based studies, this review efficiently screens out experimental intervention research, thereby retaining observational studies that are more likely to utilize analytical techniques such as regression analysis or structural equation modeling to investigate complex relationships among variables.

Following de-duplication, two researchers independently screened studies against the inclusion/exclusion criteria. They firstly assessed titles and abstracts to identify papers for full-text retrieval. Beyond studies meeting criteria after full-text review, we also scrutinized reference lists of included articles and relevant systematic reviews to identify potentially eligible studies. The researchers cross-verified their selections, resolving any discrepancies through consultation with a third researcher for a final decision.

### 3.3 Quality assessment of the included studies

**FIGURE 1:**
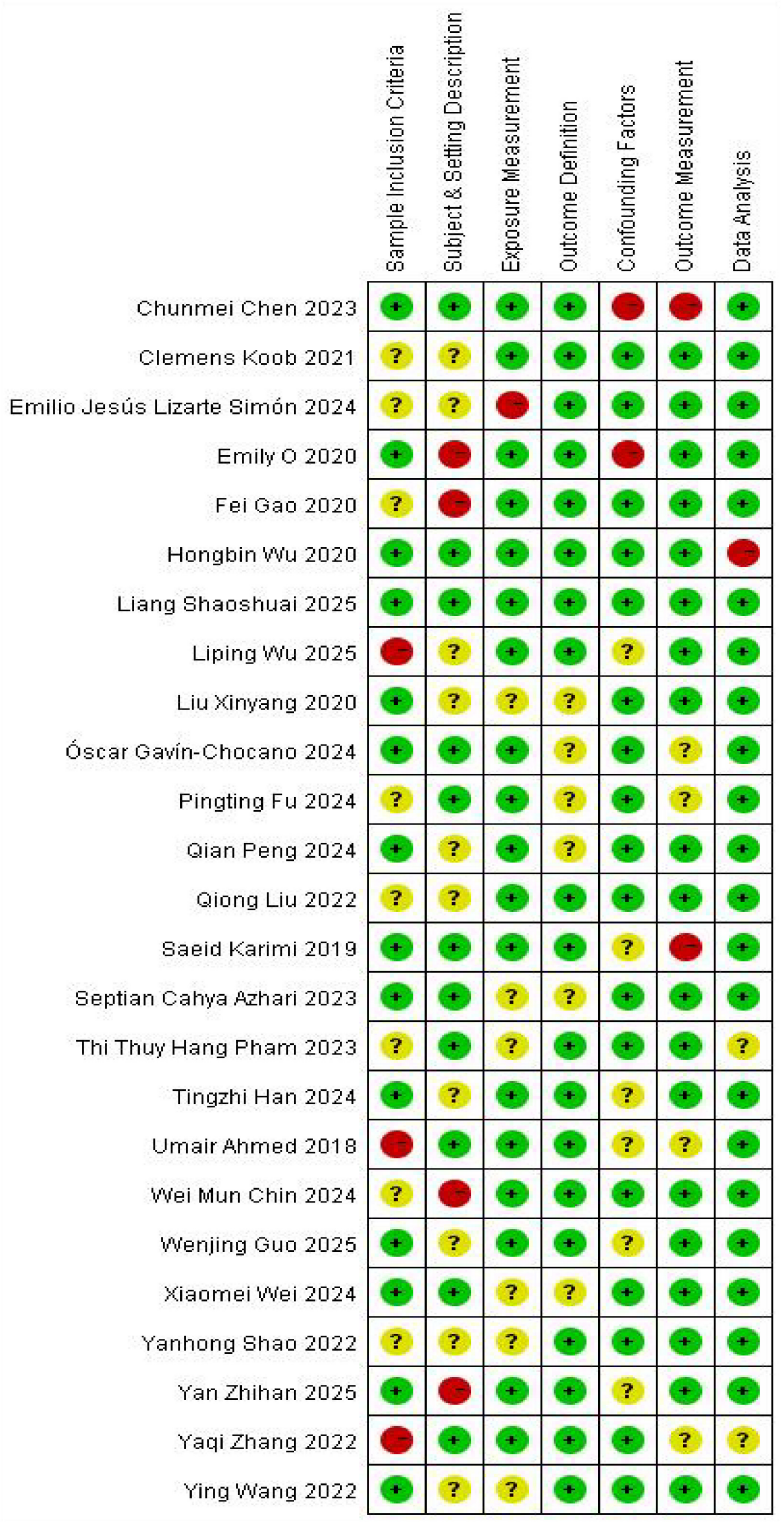
Studies’ quality assessment results.

The methodological quality of the 25 included studies was assessed using the JBI Critical Appraisal Tool for Analytical Cross-Sectional Studies (originally “JBI Evidence-Based Health Care Center Quality Assessment Tool for Analytical Cross-Sectional Studies”). Study quality scores exhibited a range of 61.2 to 100% and a mean of 72.4%. Quality assessments categorized 13 studies (52%) as excellent, 8 (32%) as good, and 4 (16%) as acceptable. No studies were rated sufficient or poor, as detailed in Figure 1.

#### 3.3.1 Data extraction

Two authors independently extracted data from included studies using a standardized form, resolving discrepancies through discussion. Extracted elements comprised: (1) author(s) and publication year; (2) study location; (3) study design; (4) psychological factor assessment methods; (5) learning engagement assessment methods; (6) statistical analyses; (7) association indicators (standardized coefficients, standard error, p-value); (8) key findings and reference. Table 1 presented comprehensive details of the journal.

**TABLE 1:** Summary of the included literature.

Number	Author/Year	Study design	Country	Sample size	Analysis	Association indicators	Conclusion	Reference
1	Pingting Fu, ChengjinGao, Xueyi Chen, Zihao Zhang, Jufeng Chen and DongYang (2024)	CS	China	1,049	Regression Analysis	β = 0.437, SE = 0.031, *P* < 0.001	Academic motivation is positively related to learning engagement.	(Fu et al., 2024)
2	Thi Thuy Hang Pham, Thi Truc Quynh Ho, Be Thi Ngoc Nguyen, Hung Thanh Nguyen and Thi Ha Nguyen (2024)	CS	Vietnam	1,638	SEM	β = 0.426, SE = 0.003, *p* < 0.01	Academic motivation is positively related to learning engagement	(Pham et al., 2024)
3	Wei Mun Chin, Nor Aniza Ahmad, Ismi Arif Ismail, Siti Noormi Alias (2024)	CS	Malaysia	461	Regression Analysis	β = 0.504, SE = 0.042, *p* < 0.01	Academic motivation is positively related to learning engagement.	(Chin et al., 2024)
4	Qian Peng, Shaoshuai Liang, Ravindran Latha, Na Li, Aiyan Zheng(2024)	CS	China	336	SEM	β = 0.334, SE = 0.048, *p* < 0.01	Academic motivation is positively related to learning engagement.	(Peng et al., 2024)
5	Yan Zhihan (2025)	CS	China	699	SEM	β = 0.972, SE = 0.28, *p* < 0.01	Academic motivation is positively related to learning engagement.	(Yan, 2025)
6	Liu Xinyang (2020)	CS	China	360	SEM	β = 0.40, SE = 0.04, *p* < 0.01	Academic motivation is positively related to learning engagement.	(Liu, 2020)
7	Saeid Karimi and Behnoosh Sotoodeh (2019)	CS	Iran	365	SEM	β = 0.236, SE = 0.054, *p* < 0.01	Academic motivation is positively related to learning engagement.	(Karimi and Sotoodeh, 2019)
8	Ying Wang and Honggang Liu (2022)	CS	Japan	561	SEM	β = 0.193, SE = 0.030, *p* < 0.01	Academic motivation is positively related to learning engagement.	(Wang and Liu, 2022)
9	Septian Cahya Azhari, Siti Fadjarajani, Ely Satiyasih Rosali (2023)	CS	Indonesia	100	SEM	β = 0.437, SE = 0.09, *p* < 0.01	Academic motivation is positively related to learning engagement.	(Azhari et al., 2023)
10	Óscar Gavín-Chocano, Inmaculada García-Martínez, Eufrasio Pérez-Navío, Antonio Luque de la Rosa (2024)	CS	Spain	648	SEM	β = 0.603, SE = 0.07, *p* < 0.01	Academic motivation is positively related to learning engagement.	(Gavín-Chocano et al., 2024)
11	Chunmei Chen, Fei Bian and Yujie Zhu (2023)	CS	China	2,106	SEM	β = 0.246, SE = 0.014, *p* < 0.01	Academic Motivation is positively related to learning engagement.	(Chen et al., 2023)
12	Hongbin Wu, Shan Li, Juan Zheng and Jianru Guo (2020)	CS	Canada	1,930	SEM	β = 0.5, SE = 0.02, p < 0.01	Academic self-efficacy plays a positively mediating role in learning engagement.	(Wu et al., 2020)
13	Tingzhi Han, Guoxing Xu, and Wenli Lu (2025)	CS	China	461	SEM	β = 0.449, SE = 0.150, *p* < 0.01	Academic self-efficacy is positively correlated with learning engagement.	(Han et al., 2025)
14	Xiaomei Wei, Nadira Saab and Wilfried Admiraal (2024)	CS	Norway	232	SEM	β = 0.166, SE = 0.063, *p* < 0.01	Academic self-efficacy is positively correlated with learning engagement.	(Wei et al., 2024)
15	Qiong Liu, Xiujun Du, Haoyu Lu (2022)	CS	China	466	SEM	β = 0.272, SE = 0.042, *p* < 0.01	Academic self-efficacy positively plays the mediating roles in learning engagement.	(Liu et al., 2022)
16	Emilio Jesús Lizarte Simón, José Gijón Puerta, María Carmen Galván Malagón and Meriem Khaled Gijón (2024)	CS	Spain	751	SEM	β = 0.22, SE = 0.06, *p* < 0.01	Academic self-efficacy positively plays the mediating roles in learning engagement.	(Simón et al., 2024)
17	Liping Wu, Yan Chen, Meiqin Xue, Weiyi Zhu and Wei Wang (2025)	CS	China	309	SEM	β = 0.469, SE = 0.188, *P* < 0.01	Academic self-efficacy positively plays the mediating roles in learning engagement.	(Wu et al., 2025)
18	Yaqi Zhang, Xiangli Guan, Md Zahir Ahmed, Mary C. Jobe and Oli Ahmed (2022)	CS	China	571	SEM	β = 0.33, SE = 0.06, *P* < 0.01	Academic self-efficacy positively plays the mediating roles in learning engagement.	(Zhang et al., 2022)
19	Liang Shaoshuai, Peng Qian, Zhang Yiwen and Xu Meiru (2025)	CS	China	428	SEM	β = 0.291, SE = 0.04, *P* < 0.01	Resilience positively plays the mediating roles in learning engagement.	(Shaoshuai et al., 2025)
20	Umair Ahmed, Waheed Ali Umrani, Muhammad Asif Qureshi, Abdul Samad (2018)	CS	Bahrain	318	SEM	β = 0.36, SE = 0.043, *P* < 0.01	Resilience is positively related to learning engagement.	(Ahmed et al., 2018)
21	Fei Gao, Qing Mei, Chenhong Guo (2020)	CS	China	374	Regression Analysis	β = 0.539, SE = 0.061, *P* < 0.01	Resilience positively plays the mediating roles in learning engagement.	(Gao et al., 2020)
22	Emily O. Adeniji, Yomi Akindele-Oscar, Sesan O. Mabekoje (2020)	CS	Nigeria	1,800	SEM	β = 0.459, SE = 0.028, *P* < 0.01	Resilience positively plays the moderating roles in learning engagement.	(Adeniji et al., 2020)
23	Yanhong Shao and Shumin Kang (2022)	CS	China	250	SEM	β = 0.307, SE = 0.064, *P* < 0.01, β = 0.367, SE = 0.075, *P* < 0.01	Academic self-efficacy and resilience positively play the mediating roles in learning engagement.	(Shao and Kang, 2022)
24	Clemens Koob, Kristina Schro pfer, Michaela CoenenID, Sandra Kus, Nicole Schmidt (2021)	CS	Germany	559	Regression Analysis	β = 0.187, SE = 0.050, *p* < 0.01, β = 0.129, SE = 0.074, *p* < 0.01	Academic self-efficacy and resilience are positively related to learning engagement.	(Koob et al., 2021)
25	WenjingGuo, JuanWang, Na Li* & Luxin Wang (2025)	CS	China	414	Regression Analysis	β = 0.232, SE = 0.045, *P* < 0.01, β = 0.340, SE = 0.047, *P* < 0.01	Academic self-efficacy and resilience positively play the mediating roles in learning engagement.	(Guo et al., 2025)

#### 3.3.2 Basic characteristics of the included studies

This systematic review includes 25 cross-sectional studies, all published since 2018, which are summarized in Table 1. The research was conducted across 12 countries in Asia (China, Malaysia, Vietnam, Japan, Indonesia, Iran, Bahrain), Europe (Spain, Norway, Germany), North America (Canada), and Africa (Nigeria), with sample sizes ranging from 100 to 2,106 participants. Methodologically, twenty studies employed structural equation modeling (SEM) and five used multiple regression, reporting standardized beta coefficients (β) and standard errors (SE) to examine the relationships between motivation, self-efficacy, psychological resilience, and learning engagement. The specific findings, methodological details, and comprehensive reference information for each study are systematically presented in Table 1 to facilitate comparison.

#### 3.3.3 Assessment instruments in included studies

The included studies employed varied instruments to measure target constructs, described below to facilitate cross-study comparison and identify potential sources of construct heterogeneity.

##### 3.3.3.1 Motivation measures

The Academic Motivation Scale (AMS) (Vallerand et al., 1992), used in three studies, is a 28-item instrument assessing intrinsic motivation, extrinsic motivation, and amotivation on a 7-point Likert scale. One study used the Study Motivation Scale (SMS) (Peng et al., 2024), examining motivational drivers linked to social norms. One applied the Work Engagement Scale (WES) (Shao and Kang, 2022). Remaining studies used adapted motivation questionnaires.

##### 3.3.3.2 Self-efficacy measures

The Academic Self-Efficacy Scale (ASES) (Chen et al., 2022), employed in four studies, evaluates learning ability self-efficacy and learning behavior self-efficacy. The MSLQ self-efficacy subscale (7 items; Han et al., 2025) assesses perceived competence on a 7-point scale. The Utrecht Work Engagement Scale-Student version (UWES-9) (Karimi and Sotoodeh, 2019) contains three subscales: vigor, dedication, and absorption. Additional measures included the Academic Efficacy Scale (Zhang et al., 2022) and a 3-item unidimensional scale (Wang et al., 2022).

##### 3.3.3.3 Psychological resilience measures

The Connor-Davidson Resilience Scale (CD-RISC) (Connor and Davidson, 2003), used in two studies, is a 25-item instrument scored on a 5-point scale assessing personal competence, tolerance of negative affect, acceptance of change, control, and spiritual influences. Other instruments included the Academic Engagement Scale with resilience subscales (Shaoshuai et al., 2025), the Study Engagement Scale (Zhang et al., 2022), and the UWES-S (Shao and Kang, 2022).

##### 3.3.3.4 Learning engagement measures

The most commonly used instrument was the UWES-S (Schaufeli et al., 2002), measuring vigor, dedication, and absorption on a 7-point frequency scale. Other measures included the Learning Engagement Scale (LES) (Heo et al., 2021) and study-specific questionnaires.

## 4 Statistical analysis

### 4.1 Rationale for analytical approach

This meta-analysis synthesizes standardized path coefficients (β) rather than bivariate correlation coefficients (r) or standardized mean differences (Cohen’s d). This decision was driven by three methodological considerations. First, 20 of 25 included studies employed SEM and 5 used multiple regression—both methods report path coefficients that account for the simultaneous influence of multiple predictors, and pooling β coefficients preserves this multivariate nature. Second, path coefficients provide estimates of each predictor’s unique contribution to learning engagement controlling for shared variance, which is more informative than bivariate associations for comparing relative predictive strength—a central objective of this study. Third, meta-analysis of path coefficients is an established approach when primary studies uniformly use regression-based methods (Cheung, 2015).

### 4.2 Effect size metric rationale

The present meta-analysis employed standardized path coefficients (β) from SEM and multiple regression analyses as the primary effect size metric. Standardized β coefficients are themselves effect sizes that allow direct comparison across studies, as they represent the expected standard deviation change in the outcome variable for a one standard deviation change in the predictor. For interpretive purposes, we adopted Cohen’s benchmarks as applied to standardized regression coefficients: β≈ 0.10 indicates a small effect, β≈ 0.30 a medium effect, and β≈ 0.50 a large effect. Fisher’s z transformation was not applied because the included studies reported path coefficients rather than bivariate correlations, and the meta-analysis was designed to synthesize the unique predictive contribution of each psychological factor as modeled in multivariate frameworks.

The majority of included studies provided data on the association between psychological factors and learning engagement in young students, along with corresponding sample sizes. For cross-sectional studies, baseline association data were extracted, whereas data from studies employing multiple assessment points were synthesized via meta-analysis. Transformed data were analyzed in Review Manager 5.4 employing fixed-effect models under conditions of low heterogeneity (*I*^2^ < 50% and *p* > 0.1) or random-effects models in cases of substantial heterogeneity (*I*^2^ ≥ 50% or *p* < 0.1); *I*^2^ values were categorized as low (≤ 25%), moderate (25–50%), or high (> 50%) in accordance with Dziri (2022) and Ilmawan (2024). Sensitivity analyses were conducted in Stata 14 to evaluate the potential impact of high heterogeneity on the pooled results (Furuya-Kanamori and Lin, 2022). Furthermore, publication bias was assessed using funnel plots and Egger’s test, with all meta-analytic results visualized by means of forest plots.

## 5 Results

### 5.1 Literature choice process

**FIGURE 2:**
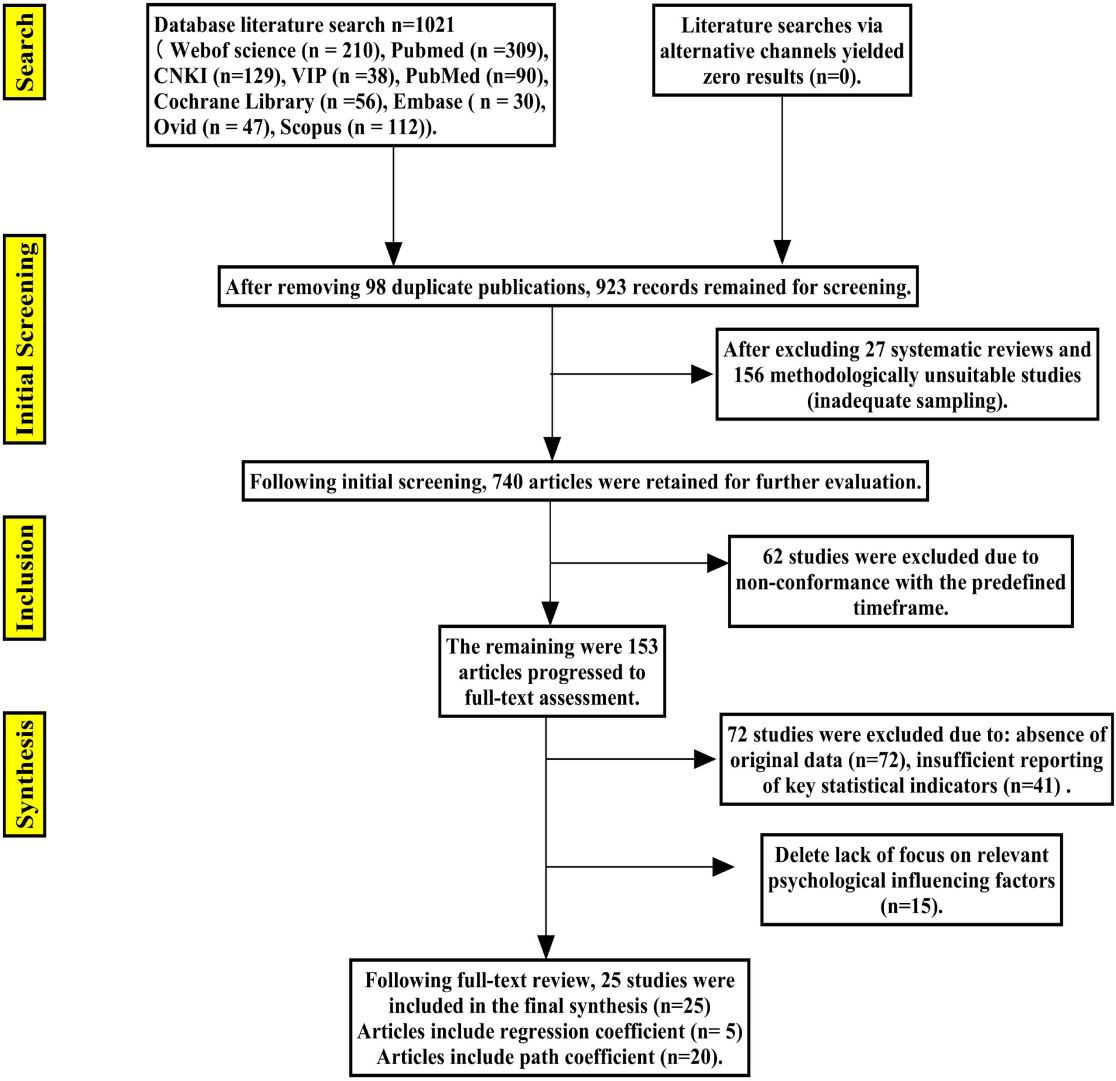
Study screening process.

Figure 2 outlines the study screening process and the rationale for exclusion. Initial database searches yielded 1,021 potentially relevant articles. Following the removal of 98 duplicates, 923 unique records were screened by title and abstract. Of these, 898 were excluded for the following reasons: they were literature reviews (*n* = 27), did not employ a relevant methodological approach or sampling strategy (*n* = 156), or fell outside the specified publication date range (*n* = 62). The remaining 153 articles underwent a full-text assessment for eligibility. At this stage, 72 studies were excluded, primarily due to an absence of original data (*n* = 72), insufficient reporting of key statistical measures (e.g., standard error; *n* = 41), or a lack of focus on the relevant psychological factors of interest, such as motivation, self-efficacy or psychological resilience (*n* = 15). Ultimately, 25 articles satisfied all inclusion criteria and were included in the review, comprising studies published from January 2018 through June 2025.

### 5.2 Meta-analysis

#### 5.2.1 Heterogeneity test

**FIGURE 3:**
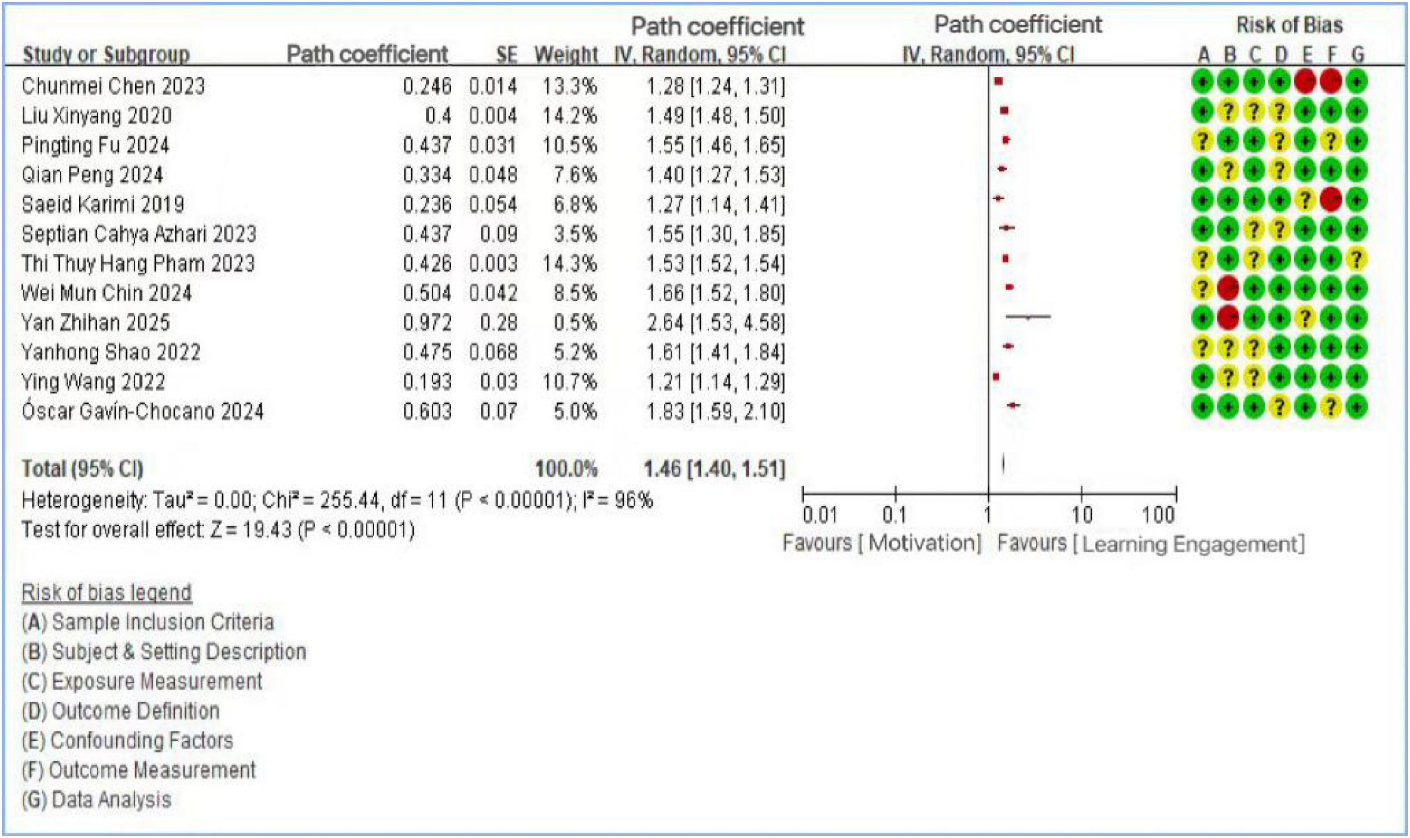
Forest plot of the relationship between motivation and learning engagement.

**FIGURE 4:**
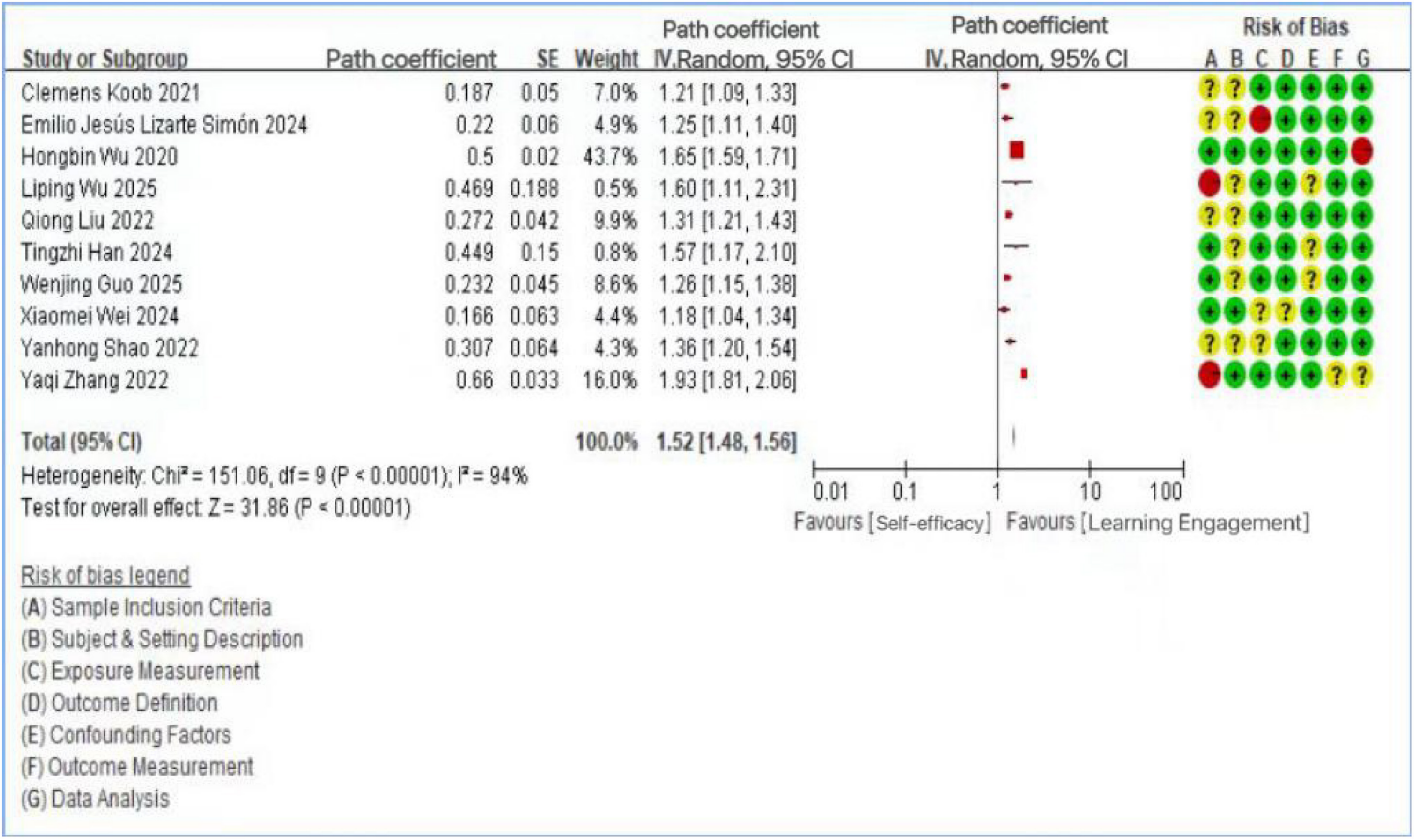
Forest plot of the relationship between self-efficacy and learning engagement.

**FIGURE 5:**
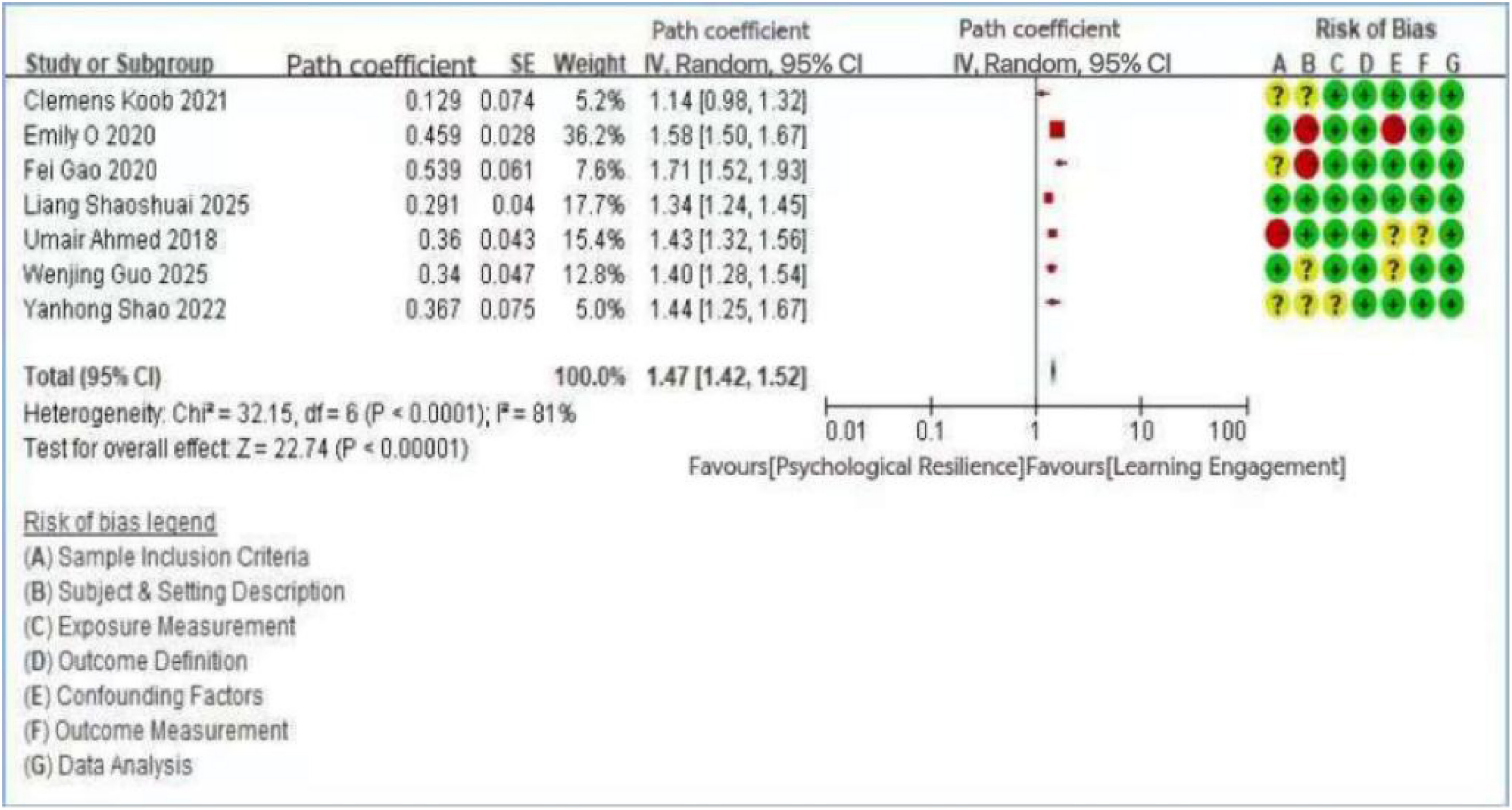
Forest plot of the relationship between psychological resilience and learning engagement.

A total of 25 articles were included in the analysis. Among these, 12 studies investigated the relationship between motivational factors and learning engagement, 10 examined the link between self-efficacy and learning engagement, and 7 explored the role of psychological resilience. It should be noted that the study by Shao and Kang (2022) included all three variables (motivation, self-efficacy, and psychological resilience), while the studies by Koob (2021) and Guo et al. (2025) included two variables each (self-efficacy and psychological resilience). Heterogeneity tests were conducted for each variable group using Review Manager, calculating standard errors (SE), path coefficients, and 95% confidence intervals. For the 12 studies on motivational factors, a random-effects model was applied due to significant heterogeneity (*I*^2^ = 96%, *p* < 0.1), prompting further sensitivity analysis. The overall effect was highly significant (*Z* = 19.43, *p* < 0.01), confirming a significant positive association—both direct and indirect—between motivational factors and learning engagement. Effect sizes ranged from β = 0.193 (Wang and Liu, 2022) to β = 0.972 (Yan, 2025), with most falling in the medium-to-large range (β > 0.30), though the substantial heterogeneity (*I*^2^ = 96%) indicates that the magnitude of this association varies considerably across studies and contexts. These findings align with research indicating that both extrinsic and intrinsic motives are essential for enhancing student engagement (Kim and Doo, 2022). Similarly, the 10 studies on self-efficacy showed substantial heterogeneity (*I*^2^ = 94%, *p* < 0.1), also necessitating sensitivity analysis. The overall effect was again significant (Z = 31.86, *p* < 0.01), supporting the view that self-efficacy is positively associated with learning engagement and may also play a mediating role in its relationship with other factors, as reported in several primary studies. Effect sizes for self-efficacy ranged from β = 0.166 (Wei et al., 2024) to β = 0.500 (Wu et al., 2020), spanning from small to large effects according to Cohen’s benchmarks. For instance, research involving Chinese college students demonstrated that academic self-efficacy is associated with learning engagement, which in turn is associated with academic achievement (Luo et al., 2023). Finally, the 7 studies on psychological resilience also revealed significant heterogeneity (*I*^2^ = 81%, *p* < 0.01) and a strong overall effect (*Z* = 22.74, *p* < 0.01), indicating both direct and indirect associations with learning engagement. Effect sizes ranged from β = 0.187 (Koob et al., 2021) to β = 0.539 (Gao et al., 2020), with the majority in the medium range. A large-scale study of 1,784 students confirmed that psychological resilience is a significant predictor of engagement levels (Li et al., 2024), while other evidence suggests that resilience may mediate the association between academic self-efficacy and engagement (Wang and Zhang, 2024). The results of the heterogeneity analyses were summarized in Figures 3–5.

#### 5.2.2 Clarification on mediation hypotheses (H3 and H5)

It is important to clarify the analytical approach to hypotheses H3 (self-efficacy as mediator) and H5 (psychological resilience as mediator). These mediation hypotheses were evaluated through a qualitative synthesis of the individual studies’ reported mediation results, rather than through a formal meta-analytic mediation analysis (e.g., two-stage structural equation modeling or Sobel test). This approach was necessitated by the fact that the primary studies did not consistently report the indirect effect estimates and bootstrap confidence intervals required for quantitative mediation meta-analysis. Among the included studies, several reported significant indirect effects of motivation on learning engagement through self-efficacy (e.g., Wu et al., 2020; Liu et al., 2022; Simón et al., 2024) and through psychological resilience (e.g., Shaoshuai et al., 2025; Gao et al., 2020). While these findings provide preliminary support for mediation pathways, they should be interpreted with caution pending formal meta-analytic structural equation modeling (MASEM) in future research.

#### 5.2.3 Sensitivity analysis

**FIGURE 6:**
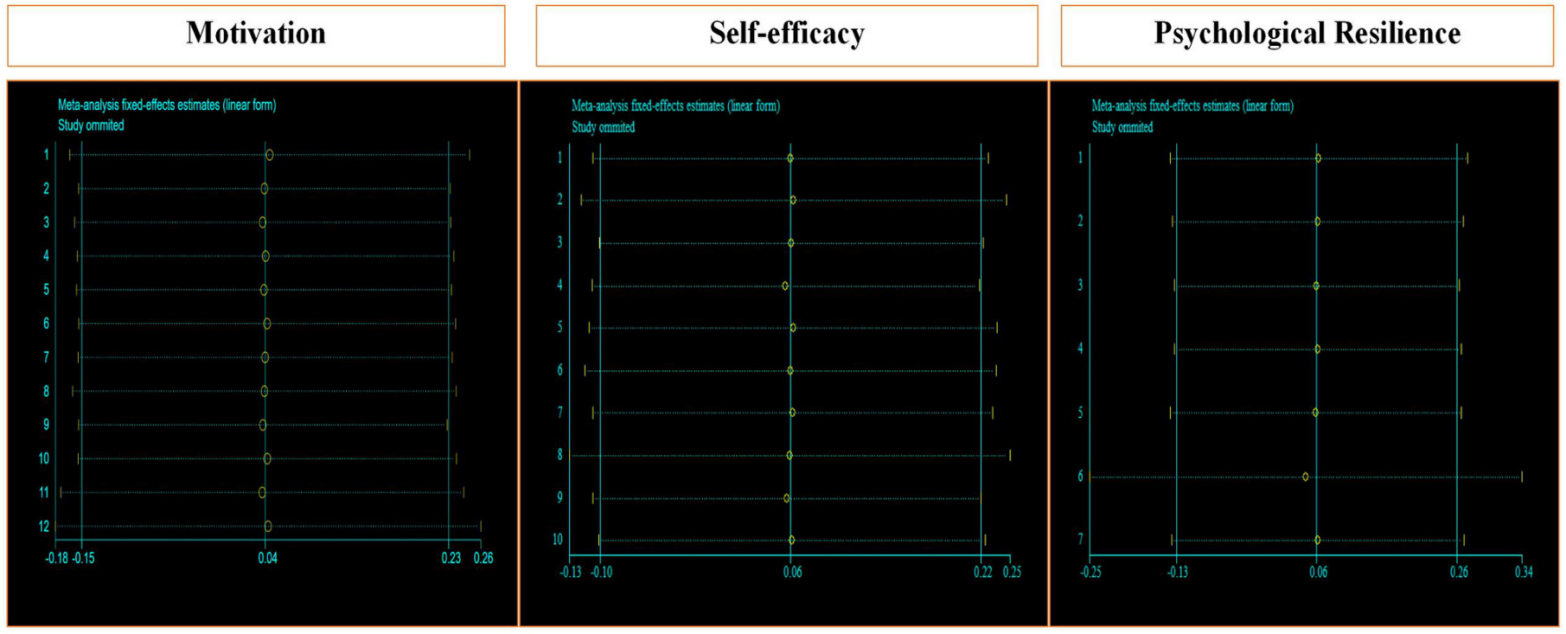
Sensitivity analysis of the relationship between motivation, self-efficacy and learning engagement.

Sensitivity analyses performed in Stata 14 across all three study groups—12 on motivational factors and learning engagement, 10 on self-efficacy and learning engagement, and 7 on psychological resilience and learning engagement—confirmed that the omission of any individual study did not substantially alter the overall meta-analytic results (Figure 6). This confirms the robustness of the findings. The dependability of the conclusions is shown by the sensitivity analyses, which verify that the results are not unduly reliant on any one study (Li and Xue, 2023). While sensitivity analyses confirm the overall robustness and stability of the meta-analytic findings, it remains important to acknowledge that individual studies may still offer unique insights worthy of deeper investigation. These nuanced perspectives could help shape future research directions, particularly given the complex interplay among these psychological constructs—an area that merits continued scholarly exploration (Navío et al., 2024).

#### 5.2.4 Publication bias assessment

**FIGURE 7:**
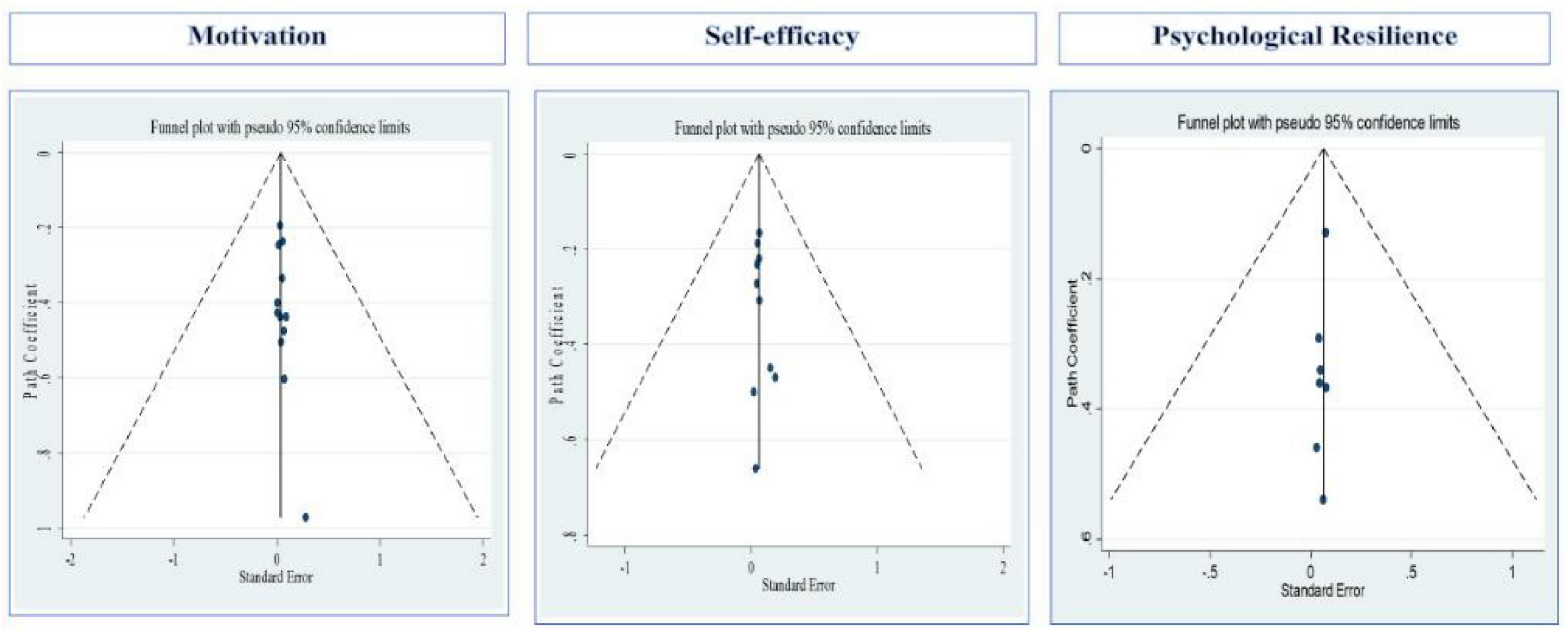
Funnel plot analyzing the relationship between motivation, self-efficacy and learning engagement.

The Egger’s test and a funnel plot were used to investigate publication bias in the included studies. In meta-analyses, the Egger’s test and funnel plot are often employed instruments to examine publication bias (Clark et al., 2017). Smaller studies with non-significant results may be underrepresented, as these techniques are intended to identify asymmetries in the distribution of study outcomes (Clark et al., 2017). The absence of publication bias in the statistical data is indicated by the symmetry of the funnel plot (Table 2 and Figure 7) and the *P*-values for the intercept coefficients from the Egger’s test for the various data types (0.076, 0.3 and 0.068). The results indicated no evidence of publication bias, as evidenced by two *p* > 0.05 and a largely symmetrical funnel plot. However, it should be noted that Egger’s test has limited statistical power when the number of included studies is small (*n* < 10 per subgroup), as is the case for the psychological resilience group (*n* = 7). Therefore, publication bias cannot be entirely ruled out, particularly given the exclusion of gray literature, including unpublished dissertations, conference proceedings, and preprints (see Limitations). These findings strengthen confidence in the meta-analysis’s integrity, as they indicate its conclusions are derived from a thorough synthesis of the available evidence (Phua et al., 2022).

**TABLE 2:** Egger’s test for motivation, self-efficacy and psychological resilience.

Egger’s test for motivation				
**Std_Eff**	**Coef.**	**Std. Err.**	***t***	***P* > t**
Slope	−0.0027508	0.0228338	−0.12	0.906
Bias	0.1332785	0.0674468	1.98	0.076
Egger’s test for self-efficacy				
**Std_Eff**	**Coef.**	**Std. Err.**	***t***	***P* > t**
Slope	0.0342608	0.0261522	1.31	0.227
Bias	0.1101046	0.0992596	1.11	0.3
Egger’s test for psychological resilience				
**Std_Eff**	**Coef.**	**Std. Err.**	***t***	***P* > t**
Slope	0.0838807	0.0101772	8.24	0
Bias	−0.0911158	0.0392056	−2.32	0.068

Std_Eff (Standardized Effect), Coef. (Coefficient), Std. Err. (Standard Error), t (*t*-statistic), P (*P*-value). Coef. indicates the direction and magnitude of the effect. Std_Eff allows comparison of effects across different variables. Std. Err. reflects the precision of the estimate. The *t* and *P* values together indicate whether the effect is statistically significant.

## 6 Study limitations and future directions

The present meta-analysis has several limitations. First, it incorporated exclusively non-randomized, cross-sectional empirical studies, with analytical metrics confined to path coefficients, standard errors, and *p*-values. Because cross-sectional designs measure variables at a single time point, temporal precedence cannot be established; all reported relationships should therefore be interpreted as associations rather than causal effects. Future longitudinal and experimental studies are essential to establish causal directionality among these constructs.

Second, the literature search was restricted to nine databases, which may have introduced publication bias and limited the comprehensiveness of the findings. Specifically, gray literature—including unpublished dissertations, conference proceedings, and preprints—was excluded, as were education-specific databases such as ERIC and broad repositories such as ProQuest and Google Scholar. No attempts were made to contact field experts or search conference proceedings for unpublished data. These omissions may have contributed to an overestimation of effect sizes. Future syntheses should broaden database coverage to include repositories such as ProQuest, Google Scholar, ERIC, and Springer.

Third, the absence of prospective protocol registration on PROSPERO or an equivalent platform represents a significant transparency limitation. Without pre-registration, it is not possible to verify whether the search strategy, inclusion criteria, or analytical plan were modified *post hoc*, limiting the ability to distinguish *a priori* from exploratory decisions. We strongly recommend that future systematic reviews in this field prospectively register their protocols.

Fourth, the extremely high heterogeneity across all three factor groups (*I*^2^ = 81–96%) warrants cautious interpretation of the pooled estimates. Several sources likely contribute to this variability: (a) heterogeneous measurement instruments across studies (e.g., AMS, SMS, WES for motivation; CD-RISC, Academic Resilience Scale for resilience), which may operationalize the same construct differently; (b) cultural and geographical differences, given that approximately 50% of included studies originated from Chinese/Asian educational contexts, where collectivistic orientations may differentially shape these psychological relationships compared to individualistic Western settings; (c) variation in sample characteristics, including age range (12–25 years) and academic discipline; and (d) differences in statistical modeling approaches (SEM vs. multiple regression). Formal subgroup analyses and meta-regression were not feasible due to insufficient reporting of moderator-level data in the primary studies.

Fifth, the exclusive focus on three internal psychological factors (motivation, self-efficacy, and psychological resilience) means that external and contextual variables—including socioeconomic status, teaching quality, class size, institutional support, peer relationships, family involvement, and technology access—were not examined. These factors may act as confounders influencing both the predictors and the outcome, potentially inflating the observed associations. Similarly, the absence of moderator analyses based on gender, age/developmental stage, academic discipline, and learning modality (online vs. face-to-face) is a notable limitation; most primary studies did not report subgroup-specific effect sizes, precluding formal moderator analyses at the meta-analytic level.

To address these gaps, future research should pursue several priorities: (a) broaden the scope of constructs to include extrinsic and contextual factors such as parent–child relationships, institutional learning climate, and peer dynamics; (b) employ meta-analytic structural equation modeling (MASEM) to formally test the mediation pathways proposed in hypotheses H3 and H5; (c) conduct instrument-based subgroup analyses to examine whether effect sizes differ systematically by measurement tool; (d) report disaggregated results by key demographic variables to enable systematic moderator analyses; (e) incorporate network meta-analysis approaches; and (f) undertake cross-cultural meta-analyses using cultural dimension frameworks (e.g., Hofstede’s indices) as moderating variables.

## 7 Discussion

This systematic review examines the associations between motivation, self-efficacy, psychological resilience, and learning engagement among young students, along with relevant influencing factors, based on 25 high-quality cross-sectional studies identified through systematic database searches; a minimum sample size of 100 participants per study was required to enhance statistical power, improve generalizability, reduce bias, and align with large-sample research standards. The analysis, conducted using RevMan and Stata 14 following data standardization, revealed statistically significant positive associations between all three psychological factors—motivation, self-efficacy, and resilience—and learning engagement, a finding further supported by recent literature such as Bahari et al. (2022), which showed that motivated students are more inclined to actively participate in learning, and Pang and Veloo (2024), who confirmed self-efficacy’s positive association with engagement and academic success. Moreover, psychological resilience proved to be a significant predictor of sustained engagement, as resilient students better manage academic demands and adversity (Li et al., 2023). Importantly, no significant publication bias was detected via funnel plot inspection or Egger’s test for path coefficients related to motivation and self-efficacy, strengthening the reliability of these findings (Furuya-Kanamori and Lin, 2022), thus providing a robust empirical foundation for further research into these psychological factors in educational contexts.

### 7.1 Comparison with previous meta-analyses

The pooled motivation effect (β = 0.42) exceeds the average motivation–engagement correlation (*r* ≈ 0.35) in Howard et al.’s (2021) meta-analysis, likely reflecting our inclusion of both intrinsic and extrinsic motivation and use of path coefficients from multivariate models. The self-efficacy effect (β = 0.31) aligns with Luo et al. (2023) and Talsma et al.’s (2018) findings of moderate self-efficacy–performance associations. The resilience effect (β = 0.36) exceeds typical resilience–academic outcome associations (Cassidy, 2016), possibly because engagement is a more proximal outcome than grades. Relative to Li and Xue’s (2023) meta-analysis of environmental factors, our larger effects likely reflect the more proximal nature of internal psychological states. However, high heterogeneity (*I*^2^ = 81–96%) indicates these pooled estimates represent central tendencies rather than fixed population values.

### 7.2 Distinguishing statistical and practical significance

It is essential to distinguish between statistical significance and practical significance in interpreting these findings. While the Z-statistics (19.43, 31.86, 22.74) confirm that the associations are statistically significant (*p* < 0.01), they do not directly indicate the magnitude or practical importance of these relationships. The standardized path coefficients reveal substantial variability: motivation β ranged from 0.193 to 0.972, self-efficacy from 0.166 to 0.500, and psychological resilience from 0.187 to 0.539. Applying Cohen’s benchmarks, some studies found only small effects (β < 0.20), while others found large effects (β > 0.50). This variability, reflected in the high I^2^ values (81–96%), suggests that the strength of these associations is highly context-dependent and that a blanket characterization of ‘strong’ relationships would oversimplify the evidence. Rather, the meta-analysis reveals statistically significant positive associations of varying magnitude, with the true effect likely differing across educational contexts, cultural settings, and measurement approaches.

### 7.3 Theoretical contributions of this study

This meta-analysis elucidates a critical nuance in academic achievement: although intelligence is a foundational predictor of success, it is not solely sufficient. The findings suggest that psychological factors—specifically motivation, self-efficacy, and psychological resilience—are significantly associated with learning engagement. Lower levels of these traits may be associated with reduced academic performance even among high-IQ students, potentially limiting the realization of their cognitive potential.

Theoretical contributions of this work are significant. As the first synthesis to investigate the integrated roles of motivation, self-efficacy, and psychological resilience concurrently, this study moves beyond examining isolated constructs. Framed within the broaden-and-build theory, it provides a consolidated empirical foundation that quantifies the significant positive associations these factors have with cognitive, behavioral, and emotional engagement. This positive association was found to be consistent across diverse educational contexts, though its magnitude varied considerably, highlighting a potential universal mechanism through which positive psychological resources may foster academic involvement and persistence (Xu et al., 2024). It offers concrete guidance for shaping future research directions and methodological approaches in this domain, enhancing our understanding of key psychological influences. Given the cross-sectional nature of all included studies, however, these findings represent associations and cannot establish causal directionality. Future longitudinal and experimental research is essential to confirm whether enhancing these psychological resources leads to improved engagement.

### 7.4 Practical implications

This study provides empirical evidence affirming significant, consistent positive associations between motivation, self-efficacy, psychological resilience, and learning engagement across diverse educational contexts. While these findings are correlational and require validation through intervention trials before widespread adoption, they yield several actionable implications for educational practice and policy.

First, educators and institutions should integrate psychological support into daily pedagogical practice. Motivation can be cultivated through Self-Determination Theory–based approaches—providing autonomy support, promoting competence, and fostering relatedness. Self-efficacy can be strengthened through Bandura’s mastery experience framework, including structured goal-setting with progressive difficulty, vicarious learning through peer modeling, and persuasive feedback. Resilience can be fostered through evidence-based programs such as the Penn Resilience Program model, typically involving 10–12 weekly sessions of approximately 90 min delivered by trained facilitators. For self-efficacy specifically, structured workshops (e.g., 8–12 weekly sessions of 60–90 min) incorporating reflective exercises, growth mindset activities, and mastery goal-setting have demonstrated effectiveness. Such interventions may be delivered by trained counselors, embedded within existing courses, or facilitated through peer mentoring programs, with evidence suggesting that sustained interventions (8–12 weeks) with regular follow-up produce more durable effects than single-session workshops.

Second, teacher training programs should include professional development modules (e.g., 2–3 day intensive workshops with ongoing mentoring) on recognizing and nurturing psychological factors that influence engagement. These modules should equip educators with practical strategies for creating motivating learning environments, delivering confidence-building feedback, and identifying students in need of additional support.

Third, successful implementation requires attention to feasibility, scalability, and cost-effectiveness. Interventions should be designed for integration into existing curricula without requiring additional class hours, recognizing potential barriers such as faculty training needs, institutional resource constraints, and varying levels of institutional commitment. Digital platforms—including online resilience-building modules and app-based self-efficacy training tools—offer promise for large-scale delivery, while peer-led and faculty-embedded approaches may reduce costs compared to specialist-delivered programs.

At the institutional level, policymakers and administrators are urged to design supportive learning environments that reduce unnecessary stressors, provide accessible mental health resources, and promote a culture valuing emotional wellbeing alongside academic achievement. Theoretical frameworks such as the broaden-and-build theory can inform the adoption of practical tools including reflective journals, resilience-building modules, and digital platforms designed to promote positive emotions and personal resources. Ultimately, this study underscores that supporting psychological development is not ancillary to academic instruction but is associated with sustaining engagement and improving educational outcomes. However, these recommendations are derived from correlational evidence, and their effectiveness should be validated through rigorously designed randomized controlled trials before large-scale implementation.

## 8 Conclusion

This study empirically validates a significant positive association between key psychological factors—motivation, self-efficacy, and psychological resilience—and learning engagement among young students, situating these relationships within the theoretical framework of the broaden-and-build theory. The findings indicate that heightened levels of these psychological resources are positively associated with learning engagement and may also be linked to indirect gains through the expansion of cognitive and behavioral repertoires and the cultivation of enduring personal resources, as posited by the theory. Consequently, enhanced engagement may serve as a potential mediator associated with improved academic performance. These results provide a robust theoretical underpinning for the design of targeted evidence-based interventions aimed at systematically strengthening these psychological factors. By integrating dedicated training programs that foster positive emotional and cognitive resources, educators may be able to promote more active and sustained student participation in learning activities, thereby leveraging engagement as an effective pathway toward optimized educational outcomes. This evidence underscores the importance of systemic integration of psychological support within educational strategies to address core psychosocial correlates of academic achievement. Given the cross-sectional nature of the included studies, all reported relationships should be interpreted as associations rather than causal effects. Future longitudinal and experimental research is needed to establish causal directionality and to validate intervention effectiveness through randomized controlled trials.

## Data Availability

Publicly available datasets were analyzed in this study. All data, analysis code, and research materials are available at https://jiaowuchu.sdctcm.edu.cn/. Our study data is available once it is requested.

## References

[B1] Adeniji E. O. Akindele-Oscar Y. Mabekoje S. O. (2020). Relationship between Family functioning and academic engagement of secondary school students: the moderating role of resilience. *Int. J. Technol. Inclusive Educ.* 9 1505–1511. 10.20533/ijtie.2047.0533.2020.0185

[B2] Ahmed U. Umrani W. A. Qureshi M. A. Samad A. (2018). Examining the links between teachers support, academic efficacy, academic resilience, and student engagement in Bahrain. *Int. J. Adv. Appl. Sc.* 5 39–46. 10.21833/ijaas.2018.09.008

[B3] Appleton J. J. Christenson S. L. Furlong M. J. (2008). Student engagement with school: critical conceptual and methodological issues of the construct. *Psychol. Schl.* 45 369–386. 10.1002/pits.20303

[B4] Azhari S. C. Fadjarajani N. S. Rosali N. E. S. (2023). The relationship between Self-Regulated learning, family support and learning motivation on students’ learning engagement. *J. Educ. Res. Eval.* 7 147–158. 10.23887/jere.v7i1.52481

[B5] Bahari G. Alharbi K. N. Alenazi L. (2022). Learning motivation and self-efficacy towards improved clinical performance in undergraduate nursing students: a cross-sectional study. *J. Clin. Diagn. Res.* 16, LC10–LC13. 10.7860/jcdr/2022/52202.15982

[B6] Bandura A. Freeman W. H. Lightsey R. (1999). Self-Efficacy: the exercise of control. *J. Cogn. Psychother.* 13 158–166. 10.1891/0889-8391.13.2.158 41472467

[B7] Cassidy S. (2016). The Academic Resilience Scale (ARS-30): a new multidimensional construct measure. *Front. Psychol.* 7:1787. 10.3389/fpsyg.2016.01787 27917137 PMC5114237

[B8] Chen C. Bian F. Zhu Y. (2023). The relationship between social support and academic engagement among university students: the chain mediating effects of life satisfaction and academic motivation. *BMC Public Health* 23:2368. 10.1186/s12889-023-17301-3 38031093 PMC10688496

[B9] Chen L. (2024). Delving into the role of self-efficacy in predicting motivation and engagement among music learners. *Learn. Motivat.* 86:101961. 10.1016/j.lmot.2024.101961

[B10] Chen P. Lin C. Lin I. Lo C. O. (2022). The Mediating Effects of Psychological capital and Academic Self-Efficacy on learning outcomes of college freshmen. *Psychol. Rep.* 126 2489–2510. 10.1177/00332941221077026 35343336

[B11] Cheung M. W.-L. (2015). *Meta-Analysis: A Structural Equation Modeling Approach.* Hoboken, NJ: Wiley. 10.1002/9781118957813

[B12] Chin W. M. Ahmad N. A. Ismail I. A. Alias S. N. (2024). Impact of student academic support on student engagement: the mediating role of basic psychological needs and academic motivation. *Asian J. Univ. Educ.* 20 53–74. 10.24191/ajue.v20i1.25740

[B13] Chu W. Yan Y. Wang H. Liu H. (2024). Visiting the studies of resilience in language learning: from concepts to themes. *Acta Psychol.* 244:104208. 10.1016/j.actpsy.2024.104208 38471348

[B14] Clark B. Frewer L. Panzone L. Stewart G. (2017). The need for formal evidence synthesis in food Policy: a case study of Willingness-to-Pay. *Animals* 7:23. 10.3390/ani7030023 28287420 PMC5366842

[B15] Connor K. M. Davidson J. R. T. (2003). Development of a new resilience scale: The Connor-Davidson Resilience Scale (CD-RISC). *Depress. Anxiety* 18, 76–82. 10.1002/da.10113 12964174

[B16] Crisostomus Y. Saraswati K. D. (2023). Pengaruh modal psikologis terhadap academic engagement (Studi Pada Mahasiswa Universitas X). *Prov. J. Psikologi Pendidikan* 16 47–54. 10.24912/provitae.v16i2.26702

[B17] Deci E. L. Ryan R. M. (2000). The “what” and “why” of goal pursuits: human needs and the self-determination of behavior. *Psychol. Inq.* 11 227–268. 10.1207/S15327965PLI1104_01

[B18] Dian-Zhi L. (2005). *The Broaden-and-Build Theory of Positive Emotions and its Enlightenment on Education.* Zhejiang: Journal of Ningbo University.

[B19] Dziri C. (2022). How to assess heterogeneity for a meta-analysis? *Tunis Med* 100:353. 36206082 PMC9552239

[B20] Fredricks J. A. Blumenfeld P. C. Paris A. H. (2004). School engagement: potential of the concept, state of the evidence. *Rev. Educ. Res.* 74 59–109. 10.3102/00346543074001059 38293548

[B21] Fredrickson B. L. (2001). The role of positive emotions in positive psychology: the broaden-and-build theory of positive emotions. *Am. Psychol.* 56 218–226. 10.1037/0003-066x.56.3.218 11315248 PMC3122271

[B22] Fu P. Gao C. Chen X. Zhang Z. Chen J. Yang D. (2024). Proactive personality and its impact on online learning engagement through positive emotions and learning motivation. *Sci. Rep.* 14:28144. 10.1038/s41598-024-79776-3 39548208 PMC11568340

[B23] Furuya-Kanamori L. Lin L. (2022). Comment on a review of methods to assess publication and other reporting biases in meta-analysis. *Res. Synth. Methods* 13 390–391. 10.1002/jrsm.1550 35118805

[B24] Gao F. Mei Q. Guo C. (2020). Relationship between depression and student engagement of senior high school students and the mediating role of resilience. *Rev. Argentina Clin. Psicol.* 29:14. 10.24205/03276716.2020.3

[B25] Gavín-Chocano Ó García-Martínez I. Pérez-Navío E. de la Rosa A. L. (2024). Learner Engagement, academic motivation and learning strategies of university students. *Educación* 27 57–79. 10.5944/educxx1.36951

[B26] Guo W. Wang J. Li N. Wang L. (2025). The impact of teacher emotional support on learning engagement among college students mediated by academic self-efficacy and academic resilience. *Sci. Rep.* 15:3670. 10.1038/s41598-025-88187-x 39881159 PMC11779917

[B27] Han T. Xu G. Lu W. (2025). Examining the effects of different types of achievement goal orientation on undergraduate students’ engagement in distance learning: the mediating effect of self-Efficacy. *Behav. Sci.* 15:39. 10.3390/bs15010039 39851843 PMC11761647

[B28] Heo H. Bonk C. J. Doo M. Y. (2021). Enhancing learning engagement during COVID-19 pandemic: self-efficacy in time management, technology use, and online learning environments. *J. Comput. Assist. Learn.* 37 1640–1652. 10.1111/jcal.12603

[B29] Howard J. L. Bureau J. S. Guay F. Chong J. X. Y. Ryan R. M. (2021). Student motivation and associated outcomes: a meta-analysis from self-determination theory. *Perspect. Psychol. Sci.* 16 1300–1323. 10.1177/1745691620966789 33593153

[B30] Ilmawan M. (2024). Navigating heterogeneity in meta-analysis: methods for identification and management. *Deka Med.* 1:e269. 10.69863/dim.2024.e269

[B31] Jiang Z. Zhang P. (2024). Does the quality of education impact students’ psychological wellbeing? Engagement as a mediator. *Soc. Behav. Pers. Int. J.* 52 1–9. 10.2224/sbp.13780

[B32] Karimi S. Sotoodeh B. (2019). The mediating role of intrinsic motivation in the relationship between basic psychological needs satisfaction and academic engagement in agriculture students. *Teach. High. Educ.* 25 959–975. 10.1080/13562517.2019.1623775

[B33] Kiken L. G. Fredrickson B. L. (2017). *Cognitive Aspects of Positive Emotions: A Broader View for Well-Being.* Cham: Springer eBooks, 157–175. 10.1007/978-3-319-58763-9_9

[B34] Kim J. Doo M. Y. (2022). The effects of motivation, career Decision-Making Self-Efficacy, and Self-Regulation on learning engagement of junior college students. *J. Coll. Stud. Dev.* 63 432–448. 10.1353/csd.2022.0036

[B35] Kline R. B. (2016). *Principles and Practice of Structural Equation Modeling*, 4th Edn. New York, NY: The Guilford Press.

[B36] Koob G. F. (2021). Drug addiction: Hyperkatifeia/negative reinforcement as a framework for medications development. *Pharmacolo. Rev.* 73, 163–201. 10.1124/pharmrev.120.000083 33318153 PMC7770492

[B37] Koob C. Schröpfer K. Coenen M. Kus S. Schmidt N. (2021). Factors influencing study engagement during the COVID-19 pandemic: a cross-sectional study among health and social professions students. *PLoS One* 16:e0255191. 10.1371/journal.pone.0255191 34314450 PMC8315536

[B38] Li J. Xue E. (2023). Dynamic interaction between student learning behaviour and learning environment: meta-analysis of student engagement and its influencing factors. *Behav. Sci.* 13:59. 10.3390/bs13010059 36661631 PMC9855184

[B39] Li L. Zhu M. Yao A. Yang J. Yang L. (2023). Daily stress, and mental health of professional degree graduate students in Chinese traditional medicine universities: the mediating role of learning career adaptation. *BMC Med. Educ.* 23:627. 10.1186/s12909-023-04614-5 37661266 PMC10476438

[B40] Li P. Jiang J. Han M. Fu S. Bai X. Wang X. (2024). The relationship between psychological resilience and learning engagement of college students: moderated mediation effects of upward social comparison and shame. *Acta Psychol.* 263:106280. 10.1016/j.actpsy.2026.106280 41616472

[B41] Liu Q. Du X. Lu H. (2022). Teacher support and learning engagement of EFL learners: the mediating role of self-efficacy and achievement goal orientation. *Curr. Psychol.* 42 2619–2635. 10.1007/s12144-022-04043-5

[B42] Liu X. (2020). *Analysis of Influencing Factors of Mathematics Learning Engagement based on Structural Equation Modeling.* Liaoning: Liaoning Normal University. 10.27212/d.cnki.glnsu.2020.000278

[B43] Luo Q. Chen L. Yu D. Zhang K. (2023). The mediating role of learning engagement between Self-Efficacy and academic achievement among Chinese college students. *Psychol. Res. Behav. Manage.* 16 1533–1543. 10.2147/prbm.s401145 37143904 PMC10153452

[B44] Navío E. P. Prieto M. G. V. Chocano ÓG. Martínez I. G. (2024). Explorando el papel de la autoeficacia y la motivación en las estrategias de aprendizaje de idiomas: un meta-análisis en educación superior (2020-2024). *Porta Linguarum Revista Interuniversitaria De Didáctica De Las Lenguas Extranjeras* 11 127–146. 10.30827/portalin.vixi.30543

[B45] Organisation for Economic Co-operation and Development [OECD] (2019). *PISA 2018 Results (Volume III): What School life Means for Students’ Lives.* Paris: OECD Publishing. 10.1787/acd78851-en

[B46] Pang H. Veloo A. (2024). The relation between learning engagement and academic self-efficacy toward academic achievement among University Students. *Qubahan Acad. J.* 4 170–183. 10.48161/qaj.v4n2a512

[B47] Peng Q. Liang S. Latha R. Li N. Zheng A. (2024). The influence of Self System Model of Motivational Development on college students’ learning engagement: a hybrid three stage Fuzzy Delphi and structural equation modeling approach. *Curr. Psychol.* 43 27762–27777. 10.1007/s12144-024-06378-7

[B48] Pham T. T. H. Ho T. T. Q. Nguyen B. T. N. Nguyen H. T. Nguyen T. H. (2024). Academic motivation and academic satisfaction: a moderated mediation model of academic engagement and academic self-efficacy. *J. Appl. Res. High. Educ.* 16 1999–2012. 10.1108/jarhe-10-2023-0474

[B49] Phua Q. S. Lu L. Harding M. Poonnoose S. I. Jukes A. To M. S. (2022). Systematic analysis of publication bias in neurosurgery meta-analyses. *Neurosurgery* 90 262–269. 10.1227/NEU.0000000000001788 35849494

[B50] Riswantyo A. T. Lidiawati K. R. (2021). The influence of self-efficacy on resilience in students who work in thesis. *Widyakala J. Pembangunan Jaya Univ.* 8:35. 10.36262/widyakala.v8i1.374

[B51] Schaufeli W. B. Salanova M. González-Romá V. Bakker A. B. (2002). The measurement of engagement and burnout: a two sample confirmatory factor analytic approach. *J. Happ. Stud.* 3 71–92. 10.1023/a:1015630930326

[B52] Setiamurti A. Salim R. M. A. Normawati M. Mufidah A. A. Mangunsong F. M. Safitri S. (2023). Factors affecting student engagement in psychology undergraduates studying online statistics courses in Indonesia. *Int. J. Cogn. Res. Sci. Eng. Educ.* 11 359–373. 10.23947/2334-8496-2023-11-3-359-373

[B53] Shao Y. Kang S. (2022). The link between Parent–Child relationship and learning engagement among adolescents: the chain mediating roles of learning motivation and academic Self-Efficacy. *Front. Educ.* 7:854549. 10.3389/feduc.2022.854549 PMC9384863 35992466

[B54] Shaoshuai L. Qian P. Yiwen Z. Meiru X. (2025). Influence of psychosocial factors and parent–student relationships on the academic engagement of TCM students: a structural equation modeling and multi-criteria decision-making framework. *Front. Psychol.* 16:1619509. 10.3389/fpsyg.2025.1619509 40771337 PMC12327497

[B55] Simón E. J. L. Puerta J. G. Malagón M. C. G. Gijón M. K. (2024). Influence of self-efficacy, anxiety and psychological well-Being on academic engagement during university education. *Educ. Sci.* 14:1367. 10.3390/educsci14121367

[B56] Talsma K. Schüz B. Schwarzer R. Norris K. (2018). I believe, therefore I achieve (and vice versa): a meta-analytic cross-lagged panel analysis of self-efficacy and academic performance. *Learn. Individ. Differ.* 61 136–150. 10.1016/j.lindif.2017.11.015

[B57] Vallerand R. J. Pelletier L. G. Blais M. R. Briere N. M. Senecal C. Vallieres E. F. (1992). The academic motivation scale: a measure of intrinsic, extrinsic, and amotivation in education. *Educ. Psychol. Meas.* 52 1003–1017. 10.1177/0013164492052004025

[B58] Wang Q. Xin Z. Zhang H. Du J. Wang M. (2022). The Effect of the Supervisor-student relationship on academic procrastination: the chainmediating role of academic self-efficacy and learning adaptation. *Int. J.Environ. Res. Public Health* 19:2621. 10.3390/ijerph19052621 35270310 PMC8909498

[B59] Wang Y. Liu H. (2022). The mediating roles of buoyancy and boredom in the relationship between autonomous motivation and engagement among Chinese senior high school EFL learners. *Front. Psychol.* 13:992279. 10.3389/fpsyg.2022.992279 36324789 PMC9620717

[B60] Wang Y. Zhang W. (2024). The relationship between college students’ learning engagement and academic self-efficacy: a moderated mediation model. *Front. Psychol.* 15:1425172. 10.3389/fpsyg.2024.1425172 39291178 PMC11407112

[B61] Weare K. (2015). *What works in Promoting Social and Emotional Well-Being and Responding to Mental Health Problems in Schools? Advice for Schools and Framework Document.* London: National Children’s Bureau.

[B62] Wei X. Saab N. Admiraal W. (2024). What rationale would work? Unfolding the role of learners’ attitudes and motivation in predicting learning engagement and perceived learning outcomes in MOOCs. *Int. J. Educ. Technol. High. Educ.* 21:5. 10.1186/s41239-023-00433-2

[B63] Wu H. Li S. Zheng J. Guo J. (2020). Medical students’ motivation and academic performance: the mediating roles of self-efficacy and learning engagement. *Med. Educ. Online* 25:1742964. 10.1080/10872981.2020.1742964 32180537 PMC7144307

[B64] Wu L. Chen Y. Xue M. Zhu W. Wang W. (2025). The effect of social support on learning engagement among Chinese nursing interns: the mediating role of self-efficacy. *BMC Nurs.* 24:995. 10.1186/s12912-025-03615-7 40691600 PMC12278551

[B65] Wu L. Ma C. (2022). An empirical study on the relationship among mental health, learning engagement, and academic self-efficacy of senior high school students. *J. Environ. Public Health* 2022:4253142. 10.1155/2022/4253142 36193414 PMC9526596

[B66] Xu X. Wu Z. Wei D. (2024). Perceived teacher support and student engagement: the chain mediating effect of basic psychological needs satisfaction and learning drive. *J. Psychol. Afr.* 34 73–79. 10.1080/14330237.2024.2311984

[B67] Yan Z. (2025). Research on the relationship between college students’ learning motivation, learning engagement, and deep learning: an empirical analysis based on structural equation modeling. *Ningbo Univ. Eng. J.* 37 118–124. 10.3969/j.issn.1008-7109.2025.01.018

[B68] Zhang Y. Guan X. Ahmed M. Z. Jobe M. C. Ahmed O. (2022). The association between University students’ achievement goal orientation and academic engagement: examining the mediating role of perceived school climate and academic self-efficacy. *Sustainability* 14:6304. 10.3390/su14106304

